# Public KAP towards COVID-19 and Antibiotics Resistance: A Malaysian Survey of Knowledge and Awareness

**DOI:** 10.3390/ijerph18083964

**Published:** 2021-04-09

**Authors:** Chee Tao Chang, Ming Lee, Jason Choong Yin Lee, Nicholas Chor Teng Lee, Tsu Yin Ng, Asrul Akmal Shafie, Kah Shuen Thong

**Affiliations:** 1 Clinical Research Centre, Hospital Raja Permaisuri Bainun, Ministry of Health Malaysia, Ipoh 30450, Malaysia; davidcct.crc@gmail.com; 2 Klinik Kesihatan Kampung Simee, Ministry of Health Malaysia, Ipoh 31400, Malaysia; 105leeming@gmail.com; 3 Perak Pharmaceutical Services Division, Ministry of Health Malaysia, Tanjung Rambutan 31250, Malaysia; jasonlcy@gmail.com; 4 Pharmacy Department, Hospital Raja Permaisuri Bainun, Ministry of Health Malaysia, Ipoh 30450, Malaysia; nlct86@gmail.com (N.C.T.L.); kahshuenthong@yahoo.com (K.S.T.); 5 Klinik Kesihatan Taiping, Perak, Ministry of Health Malaysia, Taiping 30400, Malaysia; tsuyinng@hotmail.com; 6 School of Pharmaceutical Sciences, University Sains Malaysia, Gelugor 11800, Malaysia

**Keywords:** COVID-19, antibiotic resistance, KAP survey model, preventive measures, Malaysia

## Abstract

This study aimed to assess the knowledge of the Malaysian public on the coronavirus disease 2019 (COVID-19) and antibiotics, the practice of preventive measures and attitude towards the new norms. The web-based questionnaire was disseminated online from 1 to 31 October 2020. Out of 2117 respondents, 1405 (66.4%) knew that transmission of COVID-19 virus could happen in asymptomatic people. In term of antibiotics knowledge, 779 (36.8%) respondents were aware that taking antibiotics could not speed up the recovery process of all infections. Less than half of the respondents (49.0%) knew that antibiotics are effective against bacterial infection only. Majority (92.3%) practiced good preventive measures. Majority of the respondents strongly agreed that quarantine should be made mandatory for all arrival from overseas (97.2%) and wearing face masks should be made mandatory in all public areas (94.0%). Respondents of Chinese ethnicity (*p* = 0.008), middle-aged (*p* = 0.002), with tertiary education (*p* = 0.015) and healthcare related education (*p* < 0.001), from the higher income groups (*p* = 0.001) were more likely to have better knowledge on COVID-19. The Malaysian public demonstrated good knowledge towards COVID-19, adequate practice of preventive measures and high acceptance towards the new norm. Knowledge on antibiotics use and resistance was poor, which warrants attention from the health authorities.

## 1. Introduction

The coronavirus disease 2019 (COVID-19) was declared as a pandemic by the World Health Organization (WHO) in March 2020. Until 15 November 2020, there were cumulatively 53.7 million confirmed cases and 1.3 million deaths reported to the WHO [1]. The COVID-19 virus is known to be transmitted through respiratory droplets produced when an infected person coughs, sneezes, talks, or breathes [2]. While COVID-19 has influenza-like symptoms, previous reports suggested it had caused higher rate of transmission, hospitalization and fatality than influenza [3].

In Malaysia, the first case of COVID-19 was reported on 25 January 2020 [4]. The number of positive cases passed the 500 mark on 16 March 2020 [4]. To contain the virus transmission, the Malaysian government implemented the Movement Control Order (MCO) on 18 March 2020 [5]. To date, Conditional Movement Control Order (CMCO) was reinforced in targeted areas with high number of COVID-19 cases [6]. The public had to adapt to the new normal including mandatory wearing of face masks at public areas, practice physical distancing, frequent hand-washing and restrictions on mass gatherings [7]. These preventive directives were widely promoted as they were reported to be effective in curbing the infection [8,9].

The knowledge, attitudes and practices (KAP) of the public are very important in this context and were widely studied after the COVID-19 outbreak [10]. In China, the general public demonstrated good knowledge, positive attitudes and appropriate practices in taking preventive measures [11]. Similar findings were found in Palestine, Saudi Arabia and Vietnam studies [12,13,14]. In contrast, lower income countries such as Syria, Pakistan, Bangladesh and Philippines reported lower KAP scores [15,16,17,18]. A local study conducted in Malaysia during the early stages of MCO found that the general public had good knowledge and positive attitudes towards the control of COVID-19, except for the practice of wearing face masks [19]. Another study in Australia reported adequate public knowledge on hand hygiene and physical distancing, but awareness on the use face mask and antibiotics remained insufficient [20]. A systematic review reported that the overall public KAP on COVID-19 was good in most countries, except a few in the initial stage of the pandemic [10].

The large increase in COVID-19 cases may lead to misuse of antibiotics. A meta-analysis of 24 studies reported that while the proportion of COVID-19 patients co-infected by bacterial source was low (6.9%), a majority of them (71.9%) received antibiotics [21]. In Malaysia, previous local studies found that the general public commonly believed antibiotics could be used to treat viral infections and expected physicians to prescribe antibiotics when they suffered from flu-like symptoms [22,23]. Such strongly held beliefs may further contribute to the misuse of antibiotics during this pandemic [24].

KAP surveys can identify knowledge gaps, cultural beliefs, or behavioural patterns that may facilitate understanding and action, as well as pose problems or create barriers development efforts. This can also serve as an important source of information for policymakers to design educational materials tailored to specific knowledge gaps among the public. While KAP studies had been conducted throughout the COVID-19 pandemic, knowledge of the general public on antibiotics use and resistance was not reported by previous COVID-19 KAP studies [10]. Moreover, most of these studies were conducted at the early stage of pandemic outbreak [11,19]. Hence, a follow-up study would be timely to measure the sustainability and changes in KAP over time. This study aims to determine the knowledge, attitude and practice related to COVID-19 and antibiotics use amongst the general public in Malaysia.

## 2. Materials and Methods

This was a cross-sectional study conducting using an online questionnaire via Google Forms. Malaysian residents were invited to take part in this survey. The link (URL) of the online questionnaire was distributed via email, mobile chat applications (e.g., WhatsApp, Telegram) and social media (e.g., Facebook, Instagram) to the target participants in October 2020 for one month. Residents who were above 18 years of age and stayed within the country since March 2020 were eligible to participate. Questions confirming the age and residential area were asked before the respondents were directed to the questionnaire page. Malaysian citizens staying permanently in other countries were excluded.

The respondents were sampled using convenience and snowball techniques. The researchers created a one-page recruitment banner and used the Ministry of Health Malaysia Facebook page as the main platform for questionnaire distribution. Personal and professional networks of the researchers were also used to maximize participation. Sample size was estimated using Raosoft online sample size calculator based on the formula for estimation of a proportion with finite population correction and using a conservative value of 50% of the population willing to wear a face mask, based on the findings by Azlan et al. [17] as the desired proportion to be estimated. This was the most conservative approach to generate the minimum sample size required for a survey-based study. With a precision of 0.025, a total of 1537 samples were required for this study.

The investigators developed the questionnaire in the national language (Malay) with reference to relevant literature [11,12,13,14,15,16,17,18,19,20,22,23]. It consisted of five sections: (i) demographic characteristics, (ii) knowledge of COVID-19 pandemic (7 items), (iii) preventive measures during COVID-19 pandemic (11 items), (iv) knowledge of antibiotics use and resistance (10 items) and v) attitude towards new norms during the COVID-19 pandemic (7 items). The responses in both the knowledge domains were categorised into “true”, “false” and “unsure”. Responses in the practice domain were categorized into “yes” or “no”. Responses in the attitude domain were measured based on a 5-point Likert scale from strongly disagree, disagree, neutral, agree to strongly agree (Appendix A).

The first draft of the questionnaire underwent face validation and content validation by one public health specialist, one infectious disease specialist and one senior clinical pharmacist to examine its (i) relevance, (ii) clarity and (iii) comprehensiveness. The content reviewers examined every question in the questionnaire draft and commented whether the question was (i) relevant and (ii) easy to understand. If the particular question was not relevant, the content reviewer will either suggest amending or removing the question which they felt problematic. If the question was unclear, the reviewer will make suggestions on paraphrasing to improve clarity. After reviewing each domain, the content reviewer then assessed the (iii) comprehensiveness, whether the important aspects within that particular domain were covered. Additional questions were suggested to improve the comprehensiveness of the domain whenever appropriate. Once satisfied, reviewers proceeded to review the subsequent domains by repeating the aforesaid steps. The suggestions from the content reviewers were then reconciled by the investigators and the questionnaire was revised accordingly before the pre-testing stage.

The questionnaire then underwent pre-testing on 10 respondents from the general public to check clarity and understanding. Comments from the respondents were reconciled and further modifications were made. A pilot test was subsequently conducted on 30 respondents to examine the reliability and validity of the questionnaire. The tool demonstrated good internal consistency, with Cronbach’s alpha values ranging from 0.712–0.861 for the domains. The questionnaire was finalised after the pilot test.

This study was registered in the National Medical Research Registry (NMRR) and ethics approval was obtained from the Medical Research Ethical Committee (MREC) before the study commenced. Participants accessed the online questionnaire through a unique URL. Prior to answering the questionnaire, participants were directed to the participant information sheet, which consisted of details regarding the study. Respondents were informed that their participations were voluntary and their responses would be confidential. Participants who did not agree to participate were directed to the exit page. Those who consented voluntarily would click “agree to participate” and directed to the online questionnaire. Each participant was expected to complete the questionnaire within 15 to 20 min. All responses were saved to a secure online database.

### Statistical Analysis

The data analysis was performed using the Statistical Package for the Social Sciences (SPSS) for Windows (version 20.0; IBM, New York, NY, USA) and with statistically significant level set at 5%. All the variables were analysed descriptively. The continuous variables were presented as means and standard deviation while the categorical variables were presented as frequency and percentages.

The responses in both the knowledge domains were re-categorised into “correct”, “incorrect” and “unsure”. Each correct response was given 1 point, and no point was given to incorrect/unsure responses. Each “yes” response in the practice domain was given 1 point. For the attitude domain, each agree or strongly agree responses were given 1 point. The minimum and maximum score range for each domain was as follows: knowledge on COVID-19 (0–7), knowledge on antibiotics (0–10), practice (0–11) and attitude (0–7). An arbitrary score of more than 80% in each domain would indicate good knowledge, appropriate practice and positive attitude.

Independent samples t-test and one-way analysis of variance (ANOVA) were used to ascribe the KAP mean scores and subsequently differences across respondents with different sociodemographic characteristics (Appendix B). Univariate logistic regression was performed initially, and variables with *p* < 0.25 were included into the multivariate logistic regression model. Multivariate logistic regression was then performed to determine the predictors of good knowledge towards COVID-19 and antibiotics, attitudes and practice. This was presented with adjusted odds ratios, 95% confidence interval and *p*-value. Pearson’s correlation was performed to determine the relationship between knowledge scores on COVID-19, knowledge scores on antibiotics, practice scores and attitude scores.

## 3. Results

Out of 2217 responses, 55 did not fulfill the inclusion criteria and 45 refused consent. 2117 responses were analysed. The average age of the respondents was 32.96 years (SD = 7.69, range = 18–68), mostly resided in the Central Malaysia (844, 40.1%). Majority of the respondents were female (1546, 73.0%), Malay (1381, 65.2%), had tertiary education (1870, 88.3%), with no medical education background (1527, 72.1%), and with no chronic medical illness (1844, 87.1%). Characteristics of respondents were described in Table 1.

### 3.1. Knowledge on COVID-19

Seven questions were used to assess the knowledge of the respondents on COVID-19. The mean knowledge score was 6.36 (SD = 0.87, range 0–7). The overall proportion of correct answers was 90.9% (6.36/7 × 100). Majority of the respondents were able to answer 6 out of 7 questions correctly (1829, 86.4%). Nevertheless, only two-thirds (1405, 66.4%) of the respondents knew that transmission of COVID-19 can happen even when a person did not develop symptoms. 343 (16.2%) were unsure whether COVID-19 virus strain can mutate over time (Table 2). Differences in COVID-19 knowledge scores were observed across genders, age groups, ethnicity, educational level, occupation, medical education, household income and region (Table 3).

Multiple logistics regression was performed subsequently. Respondents within the 30–49 age group (vs. 18–29 age group, OR: 1.56, CI: 1.17–2.08, *p* = 0.002), Chinese (vs. Malay, OR = 1.77, CI: 1.16–2.70, *p* = 0.008), those with tertiary (vs. primary, OR = 6.76, CI: 1.46–31.27, *p* = 0.015) and medical education (vs. no medical education, OR = 1.92, CI: 1.34–2.75, *p* < 0.001), people with high household income (vs. low household income, OR: 2.82, CI: 1.53–5.19, *p* = 0.001) obtained higher knowledge scores. Meanwhile, patients of Indian ethnicity (vs. Malay, OR: 0.37, CI: 0.23–0.59, *p* < 0.001) scored significantly lower (Table 4).

### 3.2. Knowledge on Antibiotics Use and Resistance

Respondents were required to answer ten questions regarding antibiotics use and antibiotics resistance. The mean score of the respondents was 6.12 (SD = 2.34, range 0–10), giving 61.2% overall proportion of correct answers (6.12/10 × 100). Majority of the respondents were not able to obtain a score of 8 or more, indicating poor knowledge towards antibiotics resistance (1430, 67.5%). 916 (43.3%) respondents falsely believed that taking antibiotics could speed up the recovery process of all infections. Less than half of the respondents (1037, 49.0%) were aware that antibiotics are effective against bacterial infection only. More than two-fifths of the respondents (876, 41.4%) were unsure whether antibiotics resistance would cause mortality (Table 5). Differences in knowledge scores on antibiotics resistance were significantly different across all demographic characteristics except gender, educational level and chronic disease status (Table 3).

The following characteristics predicted higher knowledge score on antibiotics use and resistance: Chinese respondents (vs. Malay, OR:2.36, CI: 1.82–3.05, *p* < 0.001), medical education (vs. no medical education, OR: 5.25, CI: 4.17–6.61, *p* < 0.001), middle household income (vs. low household income, OR:1.68, CI:1.32–2.14, *p* < 0.001), high household income (vs. low household income, OR: 2.41, CI: 1.75–3.31, *p* < 0.001). In contrast, those worked in the private sectors (vs. government, OR: 0.76, CI: 0.59–0.98, *p* = 0.033), students (vs. government, OR: 0.58, CI: 0.37–0.91, *p* = 0.018) and unemployed (vs. government, OR: 0.66, CI: 0.45–0.97, *p* = 0.034) scored significantly lower in this domain (Table 4).

### 3.3. Practice of Preventive Measures

The practices of preventive measures during COVID-19 were measured using 11 questions. The mean practice score was 10.42 (SD = 1.26, range: 0–11), giving an overall 94.7% of good practices (10.42/11 × 100). Majority of the respondents (1953, 92.3%) practiced at least 9 preventive measures during the COVID-19 pandemic. The two preventive measures that were least practiced were hand washing for at least 20 s (1796, 84.8%) and hand washing before touching face (1861, 87.9%) (Table 6).

The practice score was associated with age, gender, occupation, healthcare related education background and region of residence (Table 3). Females (vs. male, OR: 1.90, CI: 1.35–2.67 *p* < 0.001), aged between 30–49 (vs. age 18–29, OR: 2.07, CI: 1.38–3.11, *p* < 0.001), those with medical education (vs. no medical education, OR: 1.89, CI: 1.23–2.90, *p* = 0.003) possessed higher practice score. Interestingly, those with middle income (vs. lower income, OR: 0.60, CI: 0.41–0.89, *p* = 0.013) and higher income (vs. lower income, OR: 0.51, CI: 0.30–0.86, *p* = 0.012) obtained lower practice scores (Table 4).

### 3.4. Attitude towards New Norm

Seven questions were asked to determine the attitude of the respondents towards the new norms during the COVID-19 pandemic. The mean score was 6.49 (SD = 0.83, Range: 0–7), indicating an overall 92.7% of positive attitude among the respondents (6.49/7 × 100). Majority of the respondents strongly agreed that quarantine should be made mandatory for all arrival from overseas (2058, 97.2%) and wearing face masks should be made mandatory in all public areas (1989, 94.0%). However, more than a quarter of the respondents (648, 30.6%) remained neutral or disagreed that working from home is productive (Table 7). Gender, occupation and region of residence were found to be associated with attitude scores (Table 3). Female respondents (vs. male, OR: 2.12, CI: 1.51–2.99, *p* < 0.001) were more likely to have a higher attitude scores, while students (vs. government, OR: 0.42, CI: 0.22–0.79, *p* = 0.008), unemployed people (vs. government, OR: 0.48, CI: 0.27–0.86, *p* = 0.014), people working in the private sectors (vs. government, OR: 0.49, CI: 0.32–0.75, *p* = 0.001), those with middle household income (vs. low income, OR: 0.59, CI: 0.41–0.86, *p* = 0.006), people resided in the Southern region (vs. Central region, OR: 0.55, CI: 0.35–0.88, *p* = 0.012) scored significantly lower (Table 4).

### 3.5. Correlations between Different Domains

The knowledge scores on COVID-19 had a significant and moderate correlation with the knowledge scores on antibiotics (r = 0.444, *p* < 0.001), and a weak but significant correlation with the practice scores (r = 0.076, *p* < 0.001) and attitudes scores (r = 0.117, *p* < 0.001). Attitude scores demonstrated a significant but weak correlation with the practice scores (r = 0.187, *p* < 0.001) (Table 8).

## 4. Discussion

A large number of COVID-19 related KAP studies were conducted during the pandemic, as it is important to gauge the effectiveness of public educational intervention by the health authorities. A timely update of the scenario at the ground is important to inform policymakers on the current gap of knowledge among the public. From this study, it was found that the general population of Malaysia had good knowledge towards COVID-19, practiced appropriate preventive measures to prevent COVID-19 and demonstrated positive attitudes towards the new norm during the pandemic. However, knowledge on antibiotics use and resistance was poor among the public.

The knowledge of the general public in Malaysia with regards to COVID-19 was high, with a mean score of 6.36 and an overall correct rate of 91%. Most of the KAP studies conducted previously reported adequate knowledge of the respondents towards COVID-19 [10,11,12]. This was consistent with a previous local study, where the overall correct responses were more than 80% [19]. However, there was a notable uncertainty regarding the asymptomatic transmission of COVID-19. The evidence of asymptomatic transmission remained debatable, as some studies suggesting lower rate of transmission from an asymptomatic patient [25,26]. This may have contributed to the difficulties among the respondents in obtaining accurate information.

In congruence with other studies, we found that respondents of Chinese ethnicity, middle-aged adults, those with tertiary education and people with higher household income were more knowledgeable regarding COVID-19 [11,12,19]. Not surprisingly, respondents with tertiary education were 6.7 times more likely to possess good knowledge towards COVID-19, which might be attributed to their higher health literacy. While it was evident that COVID-19 patients with pre-existing comorbidities had higher risk of mortality [27], surprisingly, the knowledge among people with chronic diseases did not differ significantly from healthy individuals. This suggested that both groups of respondents had gained COVID-19 related information over time through regular mass education campaigns.

Overall, the respondents had low antibiotics-related knowledge with a mean score of 6.1 out of 10. Notably, more than two-fifths (43%) of the respondents believed that taking antibiotics can speed up the recovery process of all infections. This was comparable to a large-scale local study, where 63% of the population believed the same [23]. Additionally, less than half (49%) of our respondents knew that antibiotics are only effective against bacteria. Similarly, previous studies reported that majority of the population believed viral infection could be cured by taking antibiotics [22,23]. The poor antibiotics-related knowledge among our respondents suggests increased risk of antibiotics misuse, which may further exacerbate global antibiotics resistance [28,29].

Respondents with higher household income had significantly better knowledge regarding antibiotics. This was in line with previous studies [30,31], as people from higher social classes were more well equipped with facilities to access health-related information. Notably, respondents with a medical-related educational background were 5 times more likely to have good knowledge score pertaining to antibiotics use and resistance [30,32,33]. This is because antibiotics-related syllabuses were commonly incorporated in training programs for healthcare providers.

Overall, the public had better knowledge towards COVID-19 than antibiotics use. Notably, information regarding COVID-19 was constantly disseminated among the general public and relevant rules were being enforced concurrently. Meanwhile, antibiotics knowledge was disseminated only through antibiotics awareness campaigns occasionally, of which the public may not aware. As there were no penalties implemented, the general public was not obliged to abide any rules of prudent antibiotics use. This may explain the higher level of knowledge towards COVID-19 in comparison with antibiotics use [7,19].

Majority of the Malaysian public demonstrated good practice of COVID-19 preventive measures. In comparison to the previous local study conducted before the MCO, practices of frequent hand washing and avoiding crowded places had increased from 80% to more than 90% over time [19]. However, 15% of the respondents did not wash their hands for more than 20 s, which was also reported in other studies [13,30].

It is noteworthy to highlight the high proportion in the practice of wearing face masks (99.5%) in contrast with other studies. Previous studies show that a large proportion of the general public did not consider wearing a face mask as a preventive measure [12,18]. Azlan and colleagues reported that only 51.2% of Malaysians wore a face mask in public areas one week before the MCO [19]. This may be attributed to the shortage of face mask supply and mixed evidence on the effectiveness of face masks in preventing COVID-19 in the early stage of the outbreak [34]. Health authorities’ messages, augmented with emergence of new evidence on the importance of face masks, may have changed the practice over time [9,35,36].

Females and middle-aged adults had higher practice scores and were more likely to practice preventive measures. This was in concordance with previous studies in China and Vietnam, where male respondents were less compliant to the practice of COVID-19 preventive measures [11,13]. Older adults were twice as likely to have good practice compared to younger adults (18–29 years old), as they may perceive the higher risk of mortality if infected by COVID-19 [37].

It is worth mentioning that higher household income was associated with lower practice scores, consistent with the findings by Azlan et al. [19]. The impact of income lost and healthcare cost incurred once diagnosed with COVID-19 may be higher among those individuals with lower income, as some relied on daily wages and temporary works [38,39]. Hence, the lower income group had an unexpected higher compliance towards COVID-19 preventive measures.

The Malaysian population, specifically females and civil servants, had positive attitudes towards most of the new norms being implemented in the country. Majority of the respondents also agreed that continuous education from the government had prepared them to face the pandemic. The Director General of Health office and the Malaysian National Security Council held daily press statements and regular press conferences regarding COVID-19 since the outbreak began [40,41]. The public were informed regarding COVID-19 daily statistics, new clusters information, preventive measures and implementation of new rules for COVID-19 prevention.

However, more than a quarter of the respondents were undecided regarding the productiveness of work from home measures. A previous survey in Malaysia reported similar findings, where 31% of the employees did not wish to continue working from home after the MCO and the productivity was affected by communication barriers and network issues [42]. Furthermore, vulnerable groups such as high school dropouts, informal workers and low-income households may involve jobs that cannot be performed from home. Therefore, the government plays a crucial role in reducing inequalities related to work from home opportunities, by enhancing human capital training and providing adequate support in technology facilities.

Notably, respondents working in the government sector possessed a higher knowledge score towards antibiotics and adopted a more positive attitude towards the new norm. This was similar to the finding by a local study conducted in the early of March 2020 [19]. This might be attributed to the high level of health literacy among the Malaysian civil servants [43]. Additionally, the civil servants also receive regular and first-hand information and directives from the government, which may further consolidate their knowledge and foster positive attitudes.

Based on our findings, the respondents’ knowledge scores on COVID-19 were significantly correlated with the practice scores and attitudes scores. Attitude scores also demonstrated a significant correlation with the practice scores. Therefore, this was consistent with the KAP framework, where good knowledge and attitudes was associated with good practice among the public. However, good knowledge scores on antibiotics were not significantly correlated with good practice scores. Hence, this suggested that the current model of public educational strategies targeted on COVID-19 knowledge may be adequate to promote prudent practice of preventive measures among the general public.

### Strength and Limitations

To our best knowledge, this was one of the first nationwide studies which assessed the knowledge of the general public towards antibiotics use and resistance after the COVID-19 outbreak, which was particularly important in the antimicrobial resistance era. The practice and attitude items covered in this study was more comprehensive than previous studies conducted in the earlier stage of COVID-19 outbreak [11,19]. We employed convenience and snowball sampling in this study through social media and the researchers’ network, hence the response rate could not be estimated. The limitation of cross-sectional study design was that the temporal relationship of outcome and exposure could not be determined. Due to the online nature of this survey, there was an under-representation of male respondents, people with primary education, those above the age of 50, and working in the private sectors, in comparison to the actual demographic distribution of Malaysian population. Therefore, our findings might not be generalized to these populations. While we asked the age of respondents before their participation, we could not ensure that no people under 18 years of age participated due to the web-based nature of the questionnaire. Future studies should employ systematic stratified sampling to ensure generalizability of the findings.

## 5. Conclusions

The Malaysian general public had good knowledge, appropriate practice and positive attitudes towards COVID-19, suggesting the effectiveness of the current educational programs provided by the health authorities. However, the majority did not have adequate knowledge regarding antibiotics use and resistance. Incorporation of antibiotics-related information in public awareness programs is warranted, especially towards the population from a lower socioeconomic class.

## Figures and Tables

**Table 1:** Demographic characteristics (*n* = 2117).

Characteristics	Frequency	Percentage
**Age (years)**		
18-29	736	34.8
30-49	1307	61.7
Above 50	74	3.5
**Gender**		
Male	571	27
Female	1546	73
**Ethnicity**		
Malay	1381	65.3
Chinese	430	20.3
Indian	117	5.5
Others	189	8.9
**Education**		
Primary or below	7	0.3
Secondary	240	11.3
Tertiary	1870	88.4
**Occupation**		
Full time (government)	730	34.5
Full time (private)	915	43.2
Student	151	7.1
Unemployed	303	14.3
Retiree	18	0.9
**Medical education background**		
Yes	590	27.9
No	1527	72.1
**Chronic medical illness**		
Yes	273	12.9
No	1844	87.1
**Household income**		
Below RM 4850	1038	49
RM 4850 to RM 10,970	793	37.5
RM 10,971 and above	286	13.5
**Region ^a^**		
Central **	844	40.1
Northern	563	26.7
Southern ****	250	11.8
Eastern ***	184	8.7
Sabah/Sarawak/Labuan	269	12.7

Note: Northern region consist of Perlis, Kedah. Pulau Pinang and Perak, ** Central region consist of Selangor, Wilayah Persekutuan and Negeri Sembilan, *** Eastern region consist of Pahang, Terengganu and Kelantan, **** Southern region consist of Melaka and Johor (Tamrin SB, Yokoyama K, Jalaludin J, Aziz NA, Jemoin N, Nordin R, Li Naing A, Abdullah Y, Abdullah M. The Association between risk factors and low back pain among commercial vehicle drivers in peninsular Malaysia: a preliminary result. Ind Health. 2007 Apr; 45(2):268–78, doi:10.2486/indhealth.45.268. PMID: 17485871). ^a^ 7 missing data, *n* = 2110.

**Table 2:** Knowledge on COVID-19 (*n* = 2117).

No.	Statement	Correct	Incorrect	Unsure
1.	The COVID-19 pandemic is of virus origin	2085 (98.5)	10 (0.5)	22 (1.0)
2.	The main clinical symptoms of COVID-19 are fever, cough, sore throat and difficulty in breathing	2102 (99.3)	5 (0.2)	10 (0.5)
3.	COVID-19 is highly contagious	2100 (99.2)	12 (0.6)	5 (0.2)
4.	Elderly, children, people with co-morbidities and immunocompromised personnel develop more complications if infected	2100 (99.2)	6 (0.3)	11 (0.5)
5.	COVID-19 virus is spread mainly through respiratory droplets.	1956 (92.4)	56 (2.6)	105 (5.0)
6.	Transmission of COVID-19 virus can only happen when a person developed symptoms	1405 (66.4)	474 (22.4)	238 (11.2)
7.	COVID-19 virus strain can mutate over time.	1725 (81.5)	49 (2.3)	343 (16.2)

**Table 3:** Univariate logistic regression for significant factors associated with knowledge on COVID-19, knowledge on antibiotics resistance, practice and attitudes score (*n* = 2117).

Variable	Knowledge on Antibiotics Resistance		Knowledge on COVID-19		Practice Scores		Attitude Scores	
	Crude OR (95% CI)	*p*	Crude OR (95% CI)	*p*	Crude OR (95% CI)	*p*	Crude OR (95% CI)	*p*
**Age, years**								
18–29	Reference		Reference		Reference		Reference	
30–49	1.43 (1.17–1.75)	<0.001	1.88 (1.46–2.42)	<0.001	1.79 (1.29–2.48)	<0.001	1.29 (0.93–1.80)	0.128
> 50	2.42 (1.49–3.93)	<0.001	1.65 (0.80–3.39)	0.173	1.64 (0.64–4.18)	0.304	1.09 (0.46–2.63)	0.834
**Gender**								
Male	Reference		Reference		Reference		Reference	
Female	1.00 (0.82–1.23)	0.975	1.05 (0.79–1.38)	0.740	2.04 (1.47–2.83)	<0.001	2.21 (1.59–3.06)	<0.001
**Ethnicity**								
Malay	Reference		Reference		Reference		Reference	
Chinese	2.94 (2.36–3.68)	<0.001	2.38 (1.59–3.57)	<0.001	1.19 (0.78–1.79)	0.404	0.73 (0.50–1.06)	0.101
Indian	1.50 (1.01–2.23)	0.044	0.48 (0.31–0.74)	0.001	2.07 (0.83–5.18)	0.119	0.84 (0.43–1.67)	0.625
Others	0.84 (0.59–1.20)	0.330	1.13 (0.72–1.77)	0.590	1.49 (0.79–2.83)	0.214	1.58 (0.78–3.18)	0.201
**Education**								
Primary or below	Reference		Reference		Reference		Reference	
Secondary	0.55 (0.06–4.76)	0.583	2.52 (0.55–11.54)	0.233	-	-	2.50 (0.28–22.13)	0.410
Tertiary	3.32 (0.39–27.63)	0.267	11.07 (2.46–49.82)	0.002	-	-	1.97 (0.24–16.46)	0.532
**Occupation**								
Government	Reference		Reference		Reference		Reference	
Private	0.44 (0.36–0.54)	<0.001	0.55 (0.39–0.75)	<0.001	0.87 (0.60–1.26)	0.465	0.49 (0.33–0.44)	0.001
Student	0.39 (0.26–0.58)	<0.001	0.48 (0.29–0.79)	0.004	0.37 (0.22–0.61)	<0.001	0.44 (0.24–0.81)	0.009
Unemployed	0.23 (0.16–0.32)	<0.001	0.39 (0.27–0.58)	<0.001	1.86 (0.98–3.54)	0.058	0.66 (0.38–1.15)	0.141
Retiree	0.73 (0.28–1.91)	0.527	0.48 (0.14–1.70)	0.256	-	-	0.88 (0.11–6.81)	0.904
**Healthcare related education**								
No	Reference		Reference		Reference		Reference	
Yes	6.64 (5.39–8.18)	<0.001	2.36 (1.69–3.30)	<0.001	1.65 (1.11–2.46)	0.014	1.33 (0.91–1.95)	0.138
**Chronic Disease**								
No	Reference		Reference		Reference		Reference	
Yes	1.11 (0.85–1.45)	0.454	1.50 (0.98–2.28)	0.057	1.01 (0.63–1.63)	0.971	0.94 (0.59–1.49)	0.787
**Household Income**								
<RM 4850	Reference		Reference		Reference		Reference	
RM 4850–RM 10,970	2.58 (2.10–3.17)	<0.001	2.19 (1.65–2.90)	<0.001	0.86 (0.61–1.22)	0.403	0.76 (0.54–1.07)	0.112
≥RM 10,971	3.55 (2.69–4.67)	<0.001	4.95 (2.78–8.82)	<0.001	0.73 (0.46–1.15)	0.174	1.05 (0.62–1.77)	0.862
**Region**								
Central	Reference		Reference		Reference		Reference	
Northern	1.63 (1.30–2.04)	<0.001	0.92 (0.67–1.27)	0.602	1.29 (0.85–1.94)	0.234	1.15 (0.75–1.75)	0.524
Southern	0.99 (0.73–1.36)	0.962	0.59 (0.40–0.85)	0.005	0.81 (0.50–1.32)	0.401	0.57 (0.37–0.89)	0.015
Eastern	1.43 (1.03–2.00)	0.036	0.74 (0.48–1.16)	0.193	0.79 (0.46–1.34)	0.375	0.85 (0.48–1.50)	0.570
Borneo	1.14 (0.84–1.53)	0.401	0.96 (0.64–1.45)	0.850	1.94 (1.03–3.63)	0.039	1.37 (0.76–2.44)	0.292

Notes: OR = odds ratio, CI = confidence interval.

**Table 4:** Multiple logistic regression for significant factors associated with knowledge on COVID-19, knowledge on antibiotics resistance, practice and attitudes score (*n* = 2117).

Variable	Knowledge on COVID-19	
**Age**		
18–29 years	Reference	
30–49 years	1.56 (1.17–2.08)	0.002
> 50 year	1.15 (0.52–2.52)	0.737
**Ethnicity**		
Malay	Reference	
Chinese	1.77 (1.16–2.70)	0.008
Indian	0.37 (0.23–0.59)	<0.001
Others	1.41 (0.88–2.25)	0.157
**Education**		
Primary or below		
Secondary	2.08 (0.44–9.74)	0.353
Tertiary	6.76 (1.46–31.27)	0.015
**Healthcare related education**		
No		
Yes	1.92 (1.34–2.75)	<0.001
**Household Income**		
<RM 4850		
RM 4850–RM 10,970	1.32 (0.96–1.82)	0.093
≥RM 10,971	2.82 (1.53–5.19)	0.001
	**Knowledge on antibiotics resistance**	
	**Adjusted OR (95%CI)**	***p***
**Ethnicity**		
Malay	Reference	
Chinese	2.36 (1.82–3.05)	<0.001
Indian	1.05 (0.67–1.64)	0.828
Others	0.88 (0.59–1.30)	0.511
**Occupation**		
Government	Reference	
Private	0.76 (0.59–0.98)	0.033
Student	0.58 (0.37–0.91)	0.018
Unemployed	0.66 (0.45–0.97)	0.034
Retiree	0.74 (0.25–2.17)	0.586
**Healthcare related education**		
No	Reference	
Yes	5.25 (4.17–6.61)	<0.001
**Household Income**		
<RM 4850	Reference	
RM 4850–RM 10,970	1.68 (1.32–2.14)	<0.001
≥RM 10,971	2.41 (1.75–3.31)	<0.001
	**Practice scores**	
**Age, years**		
18–29	Reference	
30–49	2.07 (1.38–3.10)	<0.001
> 50	1.87 (0.63–5.57)	0.262
*Gender*		
Male	Reference	
Female	1.90 (1.35–2.67)	<0.001
**Medical education**		
No	Reference	
Yes	1.89 (1.23–2.90)	0.003
**Household Income**		
<RM 4850		
RM 4850–RM 10,970	0.60 (0.41–0.89)	0.013
≥RM 10,971	0.51 (0.30–0.86)	0.012
	**Attitude Scores**	
**Gender**		
Male	Reference	
Female	2.12 (1.51–2.99)	<0.001
**Occupation**		
Government	Reference	
Private	0.49 (0.32–0.75)	0.001
Student	0.42 (0.22–0.79)	0.008
Unemployed	0.48 (0.27–0.86)	0.014
Retiree	0.98 (0.13–7.71)	0.985
**Household Income**		
<RM 4850	Reference	
RM 4850–RM 10,970	0.59 (0.41–0.86)	0.006
≥RM 10,971	0.82 (0.47–1.41)	0.472
**Region**		
Central		
Northern	1.01 (0.65–1.56)	0.970
Sothern	0.55 (0.35–0.88)	0.012
Eastern	0.77 (0.43–1.38)	0.381
Borneo	1.17 (0.64–2.11)	0.613

Notes: OR = odds ratio, CI = confidence interval; Backward stepwise multiple logistic regression analysis. Multicollinearity and interaction term were checked and not found. The Hosmer–Lemeshow test, Nagelkerke classification table and area under the curve were applied to check model fitness. Knowledge on Covid-19: Hosmer–Lemeshow test: 0.579, Nagelkerke: 0.139, Area under the curve: 86.6; Knowledge on antibiotics resistance: Hosmer–Lemeshow test: <0.001; Nagelkerke test: 0.294; Area under the curve: 77.1; Attitude: Hosmer–Lemeshow test: 0.290, Nagelkerke test: 0.055, Area under the curve: 92.3; Practice: Hosmer–Lemeshow test: 0.991; Nagelkerke test: 0.068; Area under the curve: 92.3.

**Table 5:** Knowledge on antibiotics use and resistance (*n* = 2117).

No.	Statement	Correct	Incorrect	Unsure
1.	Bacteria strains can mutate rapidly over time	1466 (69.3)	166 (7.8)	485 (22.9)
2.	Development of new antimicrobials/vaccinations is simple and does not take up much time.	1754 (82.9)	174 (8.2)	189 (8.9)
3.	Taking antibiotic can prevent all infection	1369 (64.7)	368 (17.4)	380 (17.9)
4.	Taking antibiotic can speed up the recovery process of all infection	779 (36.8)	916 (43.3)	422 (19.9)
5.	Antibiotic dosage dose adjustment can be done without consultation from the professional medical practitioners	1962 (92.6)	50 (2.4)	105 (5.0)
6.	Antibiotics is effective against bacterial infection only	1037 (49.0)	540 (25.5)	540 (25.5)
7.	Antibiotic resistance can cause mortality	1241 (58.6)	110 (5.2)	766 (36.2)
8.	Like COVID-19, resistant bacteria strain can cause similar pandemic events	1089 (51.4)	181 (8.6)	847 (40.0)
9.	Misuse of antibiotics will accelerate the antibiotic resistance process	1261 (59.6)	145 (6.8)	711 (33.6)
10.	Hand hygiene is essential to prevent antibiotic resistance.	1001 (47.3)	512 (24.2)	604 (28.5)

**Table 6:** Practice of preventive measures during the coronavirus disease 2019 (COVID-19) pandemic (*n* = 2117).

No.	Statement	Yes	No
1.	Frequent hand washing after in contact with frequent touched surfaces.	2028 (95.8)	89 (4.2)
2.	Wash hand before and after touching eyes, nose and mouth	1861 (87.9)	256 (12.1)
3.	Wash hand with water and soap or sanitizer	2093 (98.9)	24 (1.1)
4.	Wash hand for at least 20 s	1796 (84.8)	321 (15.2)
5.	Wear face mask in public area	2107 (99.5)	10 (0.5)
6.	Close mouth and nose when sneezing or coughing	2097 (99.1)	20 (0.9)
7.	Always bring along sanitizer or wet wipes	1942 (91.7)	175 (8.3)
8.	Always maintain physical distancing at least 1 m from others	2034 (96.1)	83 (3.9)
9.	Avoid crowded and narrow places	2048 (96.7)	69 (3.3)
10.	Avoid chatting and speaking at close distance	2005 (94.7)	112 (5.3)
11.	Limit physical contact: no handshake policy, greeting with hand on the chest.	2041 (96.4)	76 (3.6)

**Table 7:** Attitude towards new norm during the COVID-19 pandemic (*n* = 2117).

No.	Statement	Strongly Disagree	Disagree	Neutral	Agree	Strongly Agree
1.	Body temperature monitoring should be practiced at all public areas	19 (0.9)	15 (0.7)	84 (4.0)	238 (11.2)	1761 (83.2)
2.	Availability of hand sanitizer in public area will encourage frequent hand cleaning	11 (0.5)	9 (0.4)	36 (1.7)	149 (7.0)	1912 (90.4)
3.	Face mask wearing should be made mandatory in all public area	12 (0.6)	7 (0.3)	18(0.9)	91 (4.3)	1989 (93.9)
4.	Work from home is productive and should be encouraged	61 (2.9)	81 (3.8)	506 (23.9)	433 (20.5)	1036 (48.9)
5.	Table distancing at restaurant should be continued	14 (0.7)	8 (0.4)	68 (3.2)	229 (10.8)	1798 (84.9)
6.	Quarantine should be made mandatory for all arrival from overseas	9 (0.4)	6 (0.3)	12 (0.6)	32 (1.5)	2058 (97.2)
7.	Continuous education from the government had helped me to face this pandemic better	21 (1.0)	10 (0.5)	75 (3.5)	177 (8.4)	1834 (86.6)

**Table 8:** Correlation matrix (Spearman) of knowledge on COVID-19, knowledge on antibiotics, attitude, practice scores.

Correlations	Knowledge on Covid-19 Scores	Knowledge on Antibiotics Scores	Practice Scores	Attitude Scores
Knowledge on Covid-19 scores	1	-	-	-
Knowledge on antibiotics scores	0.444 *	1	-	-
Practice scores	0.076 *	0.026 (*p* = 0.240)	1	-
Attitude scores	0.117 *	0.012 (*p* = 0.580)	0.187 *	1

* *p* < 0.001.

## Data Availability

The dataset used in this study can be obtained from the authors upon reasonable requests.

## Appendix A

Survey tool

*Please √ at your response*

**Section A: Demographic Characteristics**

**Age: Years:**	**Postcode:**
Gender: - Male - Female	Are you a healthcare provider (e.g., doctor, nurse) or (bio)medical student? - Yes - No
Race: - Malay - Chinese - Indian - Others	Do you suffer from any chronic illness or poor medical condition? (e.g., respiratory disease, heart disease, metabolic disorders such as diabetes, (previous) cancer treatment or otherdiseases requiring chronic medication) - Yes - No
Highest Academic achievement: - Primary level - Secondary level - Tertiary level	How did you know about this survey? - Social media (Facebook, Twitter, Instagram etc.) - Instant messaging (WhatsApp) - Email - Others
Are you currently employed? - Full time - Part-time - Unemployed - Retiree (private) - Government pensioner	Monthly household income: - <RM 4850 - RM 4501–RM 10,959 - >RM 10,959

**Section B: Background Knowledge about COVID-19 Pandemic**

**No.**	**Statements**	**True**	**False**	**Unsure**
●	The COVID-19 pandemic is virus origin			
●	The main clinical symptoms of COVID-19 are fever, cough, sore throat and difficulty breathing			
●	COVID-19 can affect anyone at any stage of life			
●	Elderly, child, people with co-morbidities and immunocompromised personnel are more susceptible to COVID-19 and develops more complications if infected			
●	COVID-19 virus is spread mainly through respiratory droplets			
●	Transmission of COVID-19 virus can only happen when a person developed symptoms			
●	COVID-19 virus strains can mutate over time			

**Section C: Preventive Measures during COVID-19 pandemic**

*Please indicate which preventive measure would you continue after the Covid-19 pandemic*

**Hand Hygiene**

**No.**	**Preventive Measures**	**Yes**	**No**
●	Frequent hand washing after in contact with frequent touched surfaces		
●	Wash your hands before and after touching eyes, nose and mouth		
●	6 steps hand washing that lasts for at least 20 s		

**Personal Care**

**No.**	**Preventive Measures**	**Yes**	**No**
●	Face mask wearing at crowded area, public transport		
●	Cough and sneeze etiquette		
●	Bringing along hand sanitizers or wipes whenever going out		

**Social Interaction**

**No.**	**Preventive Measures**	**Yes**	**No**
●	Physical distancing at least 1 m		
●	Avoid crowded places		
●	Avoid talking in close distance		
●	Limit physical contact: no handshake policy, *salam letak tangan di dada*		

**Section D: Knowledge towards Antibiotic Use and Antibiotic Resistance**

**No.**	**Statement**	**Yes**	**No**	**Unsure**
**General knowledge**				
●	Bacteria strains can mutate rapidly in a short period of time			
●	Developments of new antimicrobials/vaccinations is simple and does not take up much time			
**Antibiotics use**				
●	Antibiotic use can prevent all infections from getting worst			
●	Antibiotic use can help to fasten the recovery process			
●	Antibiotic dosage adjustment can be done based on severity of disease without seeking professional medical advice			
●	Antibiotic is used for bacterial infection only			
**Antibiotics resistance**				
●	Antibiotic resistance can cause death			
●	Like COVID-19, a new resistant bacteria strain can cause similar or worst pandemic events			
●	Misuse of antibiotics will accelerate the antibiotic resistance process			
●	Hand hygiene practice is essential to prevent antibiotic resistance			

**Section E: Adapting to a new norm Post COVID-19 pandemic**

**No.**	**Question**	**Strongly Disagree**	**Disagree**	**Neutral**	**Agree**	**Strongly Agree**
●	Temperature screening should be continued at public areas and crowded areas					
●	Preparing more hand sanitizers at public areas will encourage frequent hand sanitizing					
●	Face mask wearing should be made mandatory to those suffering from respiratory tract infections					
●	Working from home is productive and should be encouraged					
●	Table distancing should be continued at food outlets					
●	Home quarantine should be made compulsory to all international travelers					
●	Continuous education on infectious disease by the government to public is essential to prevent a new outbreak					

## Appendix B

**Table A1:** Comparison of demographic characteristics with knowledge, practice and attitudes score (*n* = 2117).

Variable	Freq (n)	Knowledge on COVID-19		Knowledge on Antibiotics Resistance		Practice of Preventive Measures		Attitude towards New Norm	
		Mean (SD)	*p*-Value	Mean (SD)	*p*-Value	Mean (SD)	*p*-Value	Mean (SD)	*p*-Value
Gender									
Male	571	6.27 (0.97)	0.002	6.12 (2.30)	0.995	10.2 (1.57)	<0.001	6.34 (1.05)	<0.001
Female	1546	6.40 (0.83)		6.12 (2.35)		10.5 (1.10)		6.55 (0.72)	
Age									
18–29	736	6.20 (0.96)	<0.001	5.79 (2.35)	<0.001	10.3 (1.47)	0.001	6.46 (0.86)	0.402
30–49	1307	6.45 (0.81)		6.26 (2.30)		10.5 (1.13)		6.51 (0.80)	
≥ 50	74	6.41 (0.74)		7.01 (2.48)		10.6 (0.94)		6.46 (0.98)	
Ethnicity									
Malay	1381	6.33 (0.85)	<0.001	5.85 (2.30)	<0.001	10.37 (1.32)	0.045	6.49 (0.80)	0.586
Chinese	430	6.59 (0.74)		7.17 (2.14)		10.46 (1.14)		6.45 (0.83)	
Indian	117	6.03 (1.05)		6.11 (2.52)		10.57 (0.92)		6.50 (0.92)	
Others	189	6.32 (1.00)		5.74 (2.30)		10.59 (1.17)		6.54 (0.97)	
Education									
Primary or below	7	5.57 (0.79)	<0.001	4.71 (2.23)	<0.001	10.71 (0.76)	0.815	6.43 (0.79)	0.919
Secondary	240	5.78 (0.98)		4.60 (2.15)		10.43 (1.35)		6.47 (0.88)	
Tertiary	1870	6.44 (0.82)		6.32 (2.29)		10.41 (1.25)		6.49 (0.82)	
Occupation									
Civil servant	730	6.54 (0.72)	<0.001	6.82 (2.26)	<0.001	10.48 (1.18)	<0.001	6.58 (0.71)	0.001
Private	915	6.29 (0.96)		5.87 (2.32)		10.39 (1.30)		6.43 (0.90)	
Student	151	6.23 (0.93)		5.68 (2.24)		9.90 (1.83)		6.36 (0.84)	
Unemployed	303	6.25 (0.82)		5.41 (2.24)		10.61 (0.83)		6.53 (0.78)	
Retiree	18	6.17 (0.86)		6.67 (2.25)		10.50 (0.79)		6.22 (1.63)	
Medical education									
Yes	590	6.58 (0.70)	<0.001	7.69 (1.94)	<0.001	10.57 (1.01)	<0.001	6.54 (0.75)	0.059
No	1527	6.28 (0.91)		5.52 (2.20)		10.36 (1.34)		6.47 (0.86)	
Chronic disease									
Yes	273	6.48 (0.74)	0.008	6.39 (2.22)	0.041	10.43 (1.21)	0.826	6.56 (0.81)	0.147
No	1844	6.35 (0.89)		6.08 (2.36)		10.41 (1.26)		6.48 (0.83)	
Household income									
<RM 4850	1038	6.17 (0.94)	<0.001	5.54 (2.28)	<0.001	10.45 (1.22)	0.232	6.47 (0.83)	0.652
RM 4850–RM 10,970	793	6.52 (0.73)		6.56 (2.28)		10.40 (1.22)		6.51 (0.81)	
≥RM 10,971	286	6.65 (0.77)		7.03 (2.18)		10.31 (1.45)		6.50 (0.87)	
Region									
Central	844	6.40 (0.80)	0.004	5.99 (2.25)	0.001	10.39 (1.19)	0.009	6.53 (0.78)	0.141
Northern	563	6.41 (0.85)		6.44 (2.40)		10.49 (1.15)		6.48 (0.76)	
Sothern	250	6.18 (1.01)		5.86 (2.34)		10.25 (1.62)		6.39 (1.01)	
Eastern	184	6.32 (0.84)		6.30 (2.33)		10.28 (1.47)		6.43 (0.99)	
Borneo	269	6.36 (0.97)		5.99 (2.50)		10.58 (1.09)		6.53 (0.80)	
Overall		6.36 (0.87)	Range: 0–7	6.12 (2.34)	Range: 0–10	10.42 (1.26)	Range: 0–11	6.49 (0.83)	Range: 0–7
